# Neurological Complications in Thyroid Surgery: A Surgical Point of View on Laryngeal Nerves

**DOI:** 10.3389/fendo.2014.00108

**Published:** 2014-07-15

**Authors:** Emanuela Varaldo, Gian Luca Ansaldo, Matteo Mascherini, Ferdinando Cafiero, Michele N. Minuto

**Affiliations:** ^1^U.O. Chirurgia 1, Department of Surgery, IRCCS AOU San Martino – IST, Genoa, Italy; ^2^Department of Surgical Sciences (DISC), University of Genoa, Genoa, Italy

**Keywords:** superior laryngeal nerve, inferior laryngeal nerve, thyroid surgery, morbidity, dysphonia, dysphagia, neuromonitoring

## Abstract

The cervical branches of the vagus nerve that are pertinent to endocrine surgery are the superior and the inferior laryngeal nerves: their anatomical course in the neck places them at risk during thyroid surgery. The external branch of the superior laryngeal nerve (EB) is at risk during thyroid surgery because of its close anatomical relationship with the superior thyroid vessels and the superior thyroid pole region. The rate of EB injury (which leads to the paralysis of the cricothyroid muscle) varies from 0 to 58%. The identification of the EB during surgery helps avoiding both an accidental transection and an excessive stretching. When the nerve is not identified, the ligation of superior thyroid artery branches close to the thyroid gland is suggested, as well as the abstention from an indiscriminate use of energy-based devices that might damage it. The inferior laryngeal nerve (RLN) runs in the tracheoesophageal groove toward the larynx, close to the posterior aspect of the thyroid. It is the main motor nerve of the intrinsic laryngeal muscles, and also provides sensory innervation to the larynx. Its injury finally causes the paralysis of the omolateral vocal cord and various sensory alterations: the symptoms range from mild to severe hoarseness, to acute airway obstruction, and swallowing impairment. Permanent lesions of the RNL occur from 0.3 to 7% of cases, according to different factors. The surgeon must be aware of the possible anatomical variations of the nerve, which should be actively searched for and identified. Visual control and gentle dissection of RLN are imperative. The use of intraoperative nerve monitoring has been safely applied but, at the moment, its impact in the incidence of RLN injuries has not been clarified. In conclusion, despite a thorough surgical technique and the use of intraoperative neuromonitoring, the incidence of neurological complications after thyroid surgery cannot be suppressed, but should be maintained in a low range.

## Background

The neurological issues that might appear after thyroid surgery are those related to lesions of motor or sensory nerves whose anatomical course is in the neck. The main structures that are jeopardized during thyroid surgery are those arising from the vagus nerve at various heights: the external branch of the superior laryngeal nerve (EB-SLN) and the inferior laryngeal nerve.

These nerves and their branches have mainly motor activity on the laryngeal muscles, being responsible for both the motility of the vocal cords and of all the distinctive features of one’s voice.

The lesion for the main trunk of these nerves, or of their smaller motor branches, is responsible for the paralysis of different laryngeal muscles that might be clinically evident as a significant impairment of the voice either in its quality and intensity.

In a tertiary care referral center, this morbidity should be maintained in a due range, according to different factors that can generally be preoperatively identified: the incidence of a permanent lesion of the inferior laryngeal nerve in a surgery performed for a benign disease should be maintained below 1%. On the opposite side, a higher incidence of permanent lesions can be expected when the indication for surgery is a malignant disease or a recurrent benign or malignant disease (up to 6%). Another factor affecting the complication rate is the surgeon’s experience with the low volume surgeons (those who perform <30 cases per year) being those more prone to have higher rates of permanent nerve damages (1).

Actually, a deep knowledge of the cervical anatomy, coming from a vast experience of thyroid surgery, is necessary to tailor the extent of every single operation according to the preoperative diagnosis, thus allowing to keep undesired and permanent post-operative consequences to a minimum.

## The Superior Laryngeal Nerve

The EB-SLN, previously called the “Galli-Curci Nerve,” has been named after the famous Italian opera singer who saw her career declining after a total thyroidectomy performed for a huge goiter although in the presence of a remarkably normal motility of the vocal cords. This event has been considered related to the lesion of this tiny motor branch (that for this reason was named after the singer) until recently, when it was clarified that Galli-Curci suffered from a physiological decline of her performances due to the normal aging process. The nerve has nevertheless maintained its name in several textbooks and papers, although the true story should be remarked, to preserve the integrity of the surgeon who performed the thyroidectomy (2, 3).

The superior laryngeal nerve originates just below the inferior ganglion (nodose ganglion) of the vagus lining below the jugular foramen. About 1.5 cm inferiorly, the nerve divides into two branches: the internal one, which pierces the thyrohyoid membrane in association with the superior thyroid artery and supplies the sensory innervation to the mucosa of the larynx, and the external one (4, 5).

The EB-SLN is about 0.8 mm wide and 8–9 cm long, it courses anteriorly and inferiorly along the inferior pharyngeal constrictor muscles and the branches of the superior thyroid artery. It then curves anteriorly and medially close to the lower edge of the thyroid cartilage and then approaches the larynx within the sterno-thyro-laryngeal triangle, in the region known to surgeons as the “space of Reeve,” whose limits are: the sternothyroid muscle superiorly, the inferior constrictor and cricothyroid muscles medially and the superior pole of the thyroid inferiorly (4, 6–8).

The surgical importance of the EB-SLN is due to the close relationship between the nerve and the superior thyroid vessels. At the level of the cricoid, it divides into two branches, entering separately at the pars recta and pars obliqua of the cricothyroid heads, respectively. In most circumstances, the EB-SLN passes well above the superior thyroid pole, but variations in its caudal extent in relation to the superior pole region have been well-described. For this reason, many anatomic classifications have been proposed (7–9). The most popular classification is the Cernea’s one, based on the distance between the intersection of the EB-SLN and the superior thyroid artery, and the superior pole of the thyroid (10, 11).

Type 1 (about 60%): the EB-SLN crosses the superior thyroid vessels at least 1 cm above the superior thyroid pole.

Type 2a (17%): the EB-SLN crosses at a distance shorter than 1 cm.

Type 2b (20%): the EB-SLN crosses below the upper limit of the thyroid.

Type Ni (3%): EB-SLN not identified (subfascial/intramuscular course).

The surgical implication of this classification system is that both 2a and 2b types are considered at risk during thyroidectomy.

Variations in the incidence of Cernea’s type were described in relation to: physical height (increased number of type 1), ethnicity (increased number in type 2 in Mexican, Chinese, and Indian ethnicities) and thyroid volume (increased number in type 2 in higher volumes) (5, 7, 8).

More recently, Friedman et al. proposed a classification based on the course of the EB-SLN with respect to the inferior constrictor muscle (8, 12). In type 1 variants, the EB-SLN descends with the superior thyroid vessels, either superficial or lateral to the inferior constrictor, until it terminates in the cricothyroid. In type 2 variants, the EB-SLN penetrates the inferior constrictor, 1 cm above the inferior and lateral edge of the thyroid cartilage. In type 3 variants, the EB-SLN penetrates the superior aspect of the inferior constrictor and then continues deep to the muscle before piercing the cricothyroid.

The EB-SLN carries motor fibers to the cricothyroid, the muscle that tilts the thyroid cartilage and tenses the vocal cord by modifying the distance between the cricoid and the thyroid cartilages. Vocal fold tension and thickness influence the frequency of the vibration, thus affecting the characteristic timbre of one’s voice.

Injury of the EB-SLN causes paralysis and/or weakness of the cricothyroid, resulting in changes in voice quality, voice projection, and production of high-pitched sounds (6–8, 13–16).

The EB-SLN is jeopardized when the surgeon is dissecting around the superior pole of the thyroid gland to ligate the superior thyroid artery. The laryngeal head of the sternothyroid muscle should be considered as a landmark for the course of the nerve on the inferior constrictor (13). In cases of large goiters or in patients with a short neck, the complete or partial division of the sternothyroid muscle is recommended because it may improve access to the superior thyroid pedicle.

The anatomical variations of this nerve demand careful blunt dissection of the superior thyroid pole, which should start from the avascular space located between the medial aspect of the superior pole and the cricothyroid, allowing a good exposure of the region where the EB-SLN might be found.

A gentle traction of the thyroid in a lateral and caudal direction may be helpful to obtain a good exposure of the superior vascular pedicle; moreover, the selective ligation of the vessels of the upper pole as close to the thyroid pole as possible is recommended. Separation of the polar vessels should occur in the inferior to superior direction, to avoid the entrapment of the EB-SLN between the medial aspect of the superior pole and the cricothyroid.

Care must be taken to avoid both the stretching of the nerve and an indiscriminate use of monopolar diathermy or energy-based devices for sealing vessels, in order to prevent iatrogenic heat injuries (7, 8, 13).

**Figure 1:**
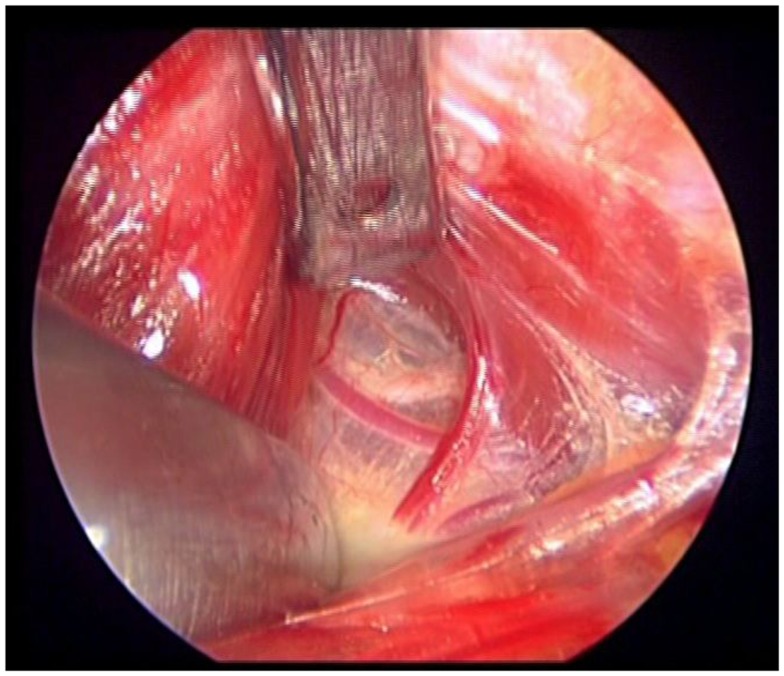
**The loop of the external branch of the superior laryngeal nerve lies very cephalic, in the “space of Reeve,” between minor vessels of the upper pedicle**. Notice that this endoscopic image is magnified 20×.

Intraoperatively, due to its wide anatomic variability, the EB-SLN can be visually recognized in some cases only (15, 17–20), and its rate of visualization during thyroid surgery is summarized in Table 1. Recently, the diffusion of minimally invasive techniques relying on the use of the endoscope has led to a higher rate of EB-SLN visualization, basically due to the magnification of the anatomy of the neck (21) (Figure 1). Finally, the use of intraoperative neuromonitoring can be useful in both the identification and preservation of the EB-SLN, as described in several reports (7, 8, 13, 15–18, 21–23).

**Table 1:** **Incidence of visualization of the external branch of the superior laryngeal nerve during thyroid surgery**.

Reference	Patients	Study	Visualization of EB	Comment
Berti et al. (21)	300	Retrospective	65%	“Incidental” visualization during MIVAT
Friedman et al. (12)	884	Retrospective	85%	Identification followed transection of the sternothyroid muscle and NM
Dionigi et al. (23)	72	Prospective, randomized	84% (NM) vs. 42% (visualization)	MIVAT
Pagedar and Freeman (18)	112	Prospective	98%	Identification followed blunt dissection of the space of Reeve
Lifante et al. (22)	47	Prospective, randomized	66% (NM) vs. 21% (no NM)	Improvement in patient-assessed voice quality after surgery but does not impact swallowing

*EB-SLN, external branch of the superior laryngeal nerve; MIVAT, minimally invasive video-assisted thyroidectomy; NM, intraoperative neuromonitoring*.

Post-operatively, patients with a lesion of the EB-SLN typically complain of voice fatigue, problems reaching high-pitch sounds that they were used to reach, and the need of an extra effort to speak; they can also complain of various rates of dysphagia.

The awareness to these symptoms, that may be subliminal to common people, might be more evident to the so-called “professional speakers,” such as singers, teachers, and lawyers.

Several studies have investigated post-operative rates of EB-SLN injury varying from 0 to as much as 58% (the most relevant experiences reported are summarized in Table 2) (24–26), a result that reflects the need for standardized protocols looking forward a more accurate evaluation of this complication, even if the impairments coming from such lesions are the less prone to be improved with specific post-operative treatments.

**Table 2:** **Incidence of lesions of the external branch of the superior laryngeal nerve during thyroid surgery: the sequence is progressive, starting from the series reporting the lowest incidence**.

Reference	Patients	Study	Visualization of EB	Rate of injury
Jonas et al. (17)	108	Prospective	37.5%	0 (nerve monitoring)
Bellantone et al. (19)	289	Prospective, randomized	0 (not searched) vs. 89% (searched)	0 vs. 0
Inabnet et al. (20)	10	Prospective	53% (out of 15 nerves at risk)	0 (nerve monitoring)
Teitelbaum et al. (24)	20	Retrospective	Not described	5%
Aluffi et al. (25)	45	Retrospective	Not described	14%
Hurtado-Lopez et al. (26)	100	Prospective, randomized	0 (not searched) vs. 78% (searched)	20% (non-visualized) vs. 8% (visualized)

*EB-SLN, external branch of the superior laryngeal nerve*.

Currently, videostroboscopy (which evaluates the regularity and symmetry of the mucosal traveling wave and the degree of glottis closure) and/or electromyography of the cricothyroid (that documents a decreased recruitment, and polyphasic action potentials that might reach the electrical silence in extreme cases) are the only instrumental tools that might allow to achieve good diagnostic standards (5, 7, 8), although both are invasive methods, responsible for some discomfort to the patients.

## The Inferior Laryngeal Nerve

The inferior laryngeal nerve (recurrent nerve, RLN) arises as the vagus courses anteriorly to the aortic arch. As the heart and the great vessels descend during the embryonic development, the RLNs are dragged down by the aortic arches. On the right side, the nerve recurs around the fourth arch (the right subclavian artery), whereas on the left, the nerve recurs around the sixth arch (the ligamentum arteriosum). The right RLN curves around the right subclavian artery and enters the base of the neck from a more lateral position, with respect to the nerve on the opposite side, a consideration that is extremely important from a strictly surgical point of view, since the anatomy is significantly different on the two different sides. The approximate length of the right RLN from the subclavian artery to the cricothyroid joint, is about 5–6 cm, whereas on the left side is about 12 cm long (from the aorta to the cricothyroid joint) (4). After leaving the superior mediastinum, the RLN runs toward the larynx in the trachea–esophageal groove, in a close anatomical relationship with the thyroid gland and the parathyroids. Immediately before or after crossing the Berry ligament, the RLN divides in smaller branches entering the esophagus (mainly sensory), and the main ones that enters the larynx at the inferior constrictor muscle, posterior to the cricothyroid joint (the motor ones).

The RLN in the neck is supplied by both its own vessels (“vasa nervorum”) and by branches of the inferior thyroid artery: this vascularization is particularly important since an excessive dissection of the nerve might theoretically represent a cause of undesired post-operative issues (4).

**Figure 2:**
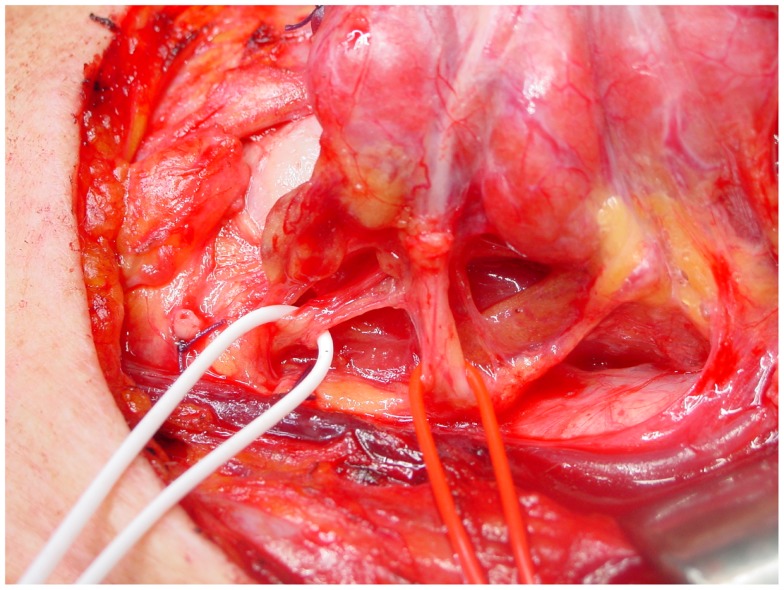
**The relationship between the inferior laryngeal nerve and the inferior thyroid artery, on the left side**. In this case, the nerve (white loop) runs posterior to the artery (red loop).

The course of the RLN with respect to inferior thyroid artery is quite variable: it more commonly courses deep to the inferior thyroid artery, but can also travel anterior to or between its branches (11, 27) (Figure 2).

Several anatomical variations of the RLN may be found: in some cases, these variations might represent an unfavorable situation and thus an additional cause of morbidity. The more common anatomical variants are: the variable relationship with the branches of the inferior thyroid artery and the ligament of Berry, the various patterns of extralaryngeal branching, and the non-recurrent variant (27).

Various anatomical relationships between the RLN and the ligament of Berry are described: although the nerve is generally located posterior and lateral to this ligament, it can also have a medial course with respect to it, and, in some cases, an anterior (motor) branch can penetrate this ligament (11, 28). This latter situation is considered the one associated with the highest surgical morbidity, since the terminal branches might be completely hidden inside the ligament, and therefore sectioned during the final part of the thyroidectomy.

Variations in the pattern of distal bifurcation of the RLN have been widely reported, since, within the larynx, the nerve divides into an external branch providing motor function to the four intrinsic laryngeal muscles, and an internal branch with only sensory activity for the glottis. This division may occur before the RLN enters the larynx near the Berry ligament or the inferior thyroid artery giving origin to up to several extralaryngeal branches extremely varying in sizes. Functional studies of the extralaryngeal nerve branches have well-demonstrated that motor branches to both the abductor and adductor muscles of the larynx are generally located in the anterior division (4, 27, 29), this adding further potential morbidity, since the motor branches are those located closest to the thyroid lobe.

**Figure 3:**
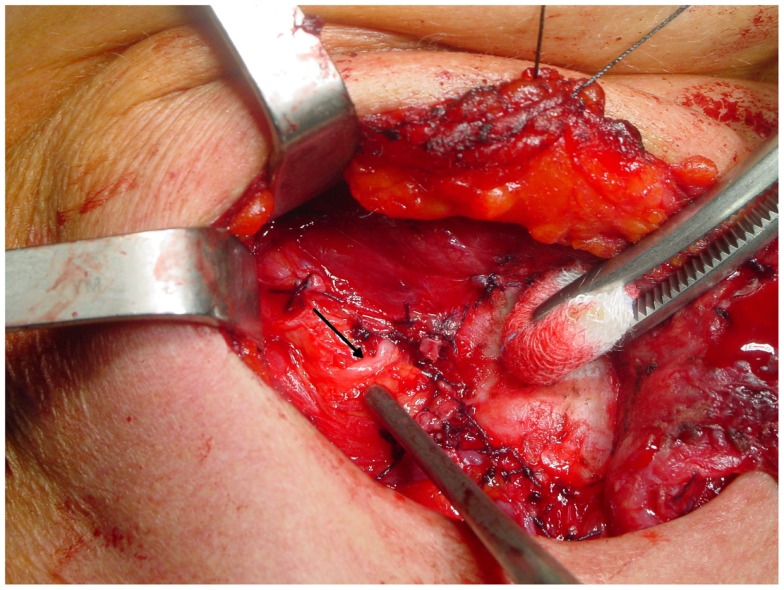
**A relatively high variant of a non-recurrent inferior laryngeal nerve on the right side: in this case, the nerve (arrow) runs almost horizontally from the vagus toward the larynx**.

The non-recurrent laryngeal nerve is associated to a major vascular variant characterized by the absence of the brachiocephalic artery and the formation of a right aberrant subclavian artery that originates from the last branch of the aortic arch and extends rightwards, running behind the esophagus (the “lusorian artery”). The nerve then originates directly from the cervical part of the vagus, at various heights, since it cannot be pulled down by the right subclavian artery during its embryological descent (Figure 3). For this reason, this variant is present almost exclusively on the right side (incidence: 0.3–1.6%), although a few cases have been described on the left side (incidence: 0.04%), mainly in patients with situs viscerum inversus (4, 29–34), since the corresponding anatomical anomaly on the left side (the absence of the ductus arteriosus) is generally incompatible with survival of the fetus. Soustelle has classified the non-recurrent laryngeal nerve into two types according to their courses. TYPE 1 arises directly from the cervical vagus and enters the larynx at the upper pole of the thyroid gland, running together with the vessels of the superior thyroid pedicle. TYPE 2 arises from the vagus nerve near the origin of the inferior thyroid artery and enters the larynx above the inferior thyroid artery (TYPE 2A), or under the inferior thyroid artery (TYPE 2B) (32, 34).

From a surgical point of view, TYPE 1 anomaly implies that the nerve could be injured during superior thyroid pedicle ligation, while TYPE 2 variant implies that, over its transverse course, the non-recurrent laryngeal nerve can mimic the course of the inferior thyroid artery, and thus be misinterpreted by the surgeon, and finally interrupted. Preoperative computed tomography showing the presence of an arteria lusoria or the presence of a “horizontal Y” at US scan (due to the bifurcation of the brachiocephalic trunk into the right common carotid artery and the right subclavian artery) could predict the presence of a non-recurrent laryngeal nerve and therefore reduce the risk of injuring it (32–34).

Transient partial RLN dysfunctions are related to segmental demyelination or focal conduction block, and transient or permanent total nerve dysfunctions result from severe myelin sheath or axonal damage and neural degeneration. Transient palsy can be regarded either as a consequence of neuroapraxia or axonotmesis, depending on whether local myelin damage, usually secondary to compression (edema, blood clot, and suffusion) or a loss of continuity of axons. An excessive manipulation during surgery might lead to a significant edema or to diffuse microhemorrhage by injury of fragile capillaries, possibly resulting in neuroapraxia or axonotmesis (29).

The final outcome of the damage to a RLN is represented by the paralysis of the sole abducting muscle (posterior cricoarytenoid) of the vocal cords, which might result in a clinical situation ranging from an impaired motility of the cord, up to its atrophy.

Consequent symptoms range from hoarseness in unilateral lesions, to stridor and acute airway obstruction in bilateral damage. Temporary or permanent post-operative vocal changes can have a serious impact on the patient’s quality of life, especially in professional voice users.

The posterior branches of the RLN often provide innervation to both the cricopharyngeus muscle and the esophagus, thus justifying the impairment in swallowing that is frequently complained by patients after thyroidectomy (31, 35–37). These branches are extremely short and thin and might be difficult to visualize and, therefore, spared by the surgeon performing the thyroidectomy. Nerve monitoring cannot obviously be used to identify such branches since they are almost exclusively sensorial.

Post-operative RLN lesion is a relatively rare complication of thyroid surgery in expert hands, the rate of permanent damages occurring approximately between 0.3 and 3% of the cases, and transient palsies in up to 8% of the cases. This wide range of results is described in Table 3 (38–46), where the most numerous studies recording the morbidity of thyroid surgery are summarized. This rate is mainly related to the diagnosis of the patient, with patients undergoing reoperative thyroid surgery and those undergoing extensive surgery for cancer being at greater risk of morbidity, and to the surgeon’s experience (1). The risk of RLN injury in reoperative surgery is described between 2 and 30%, with rate that is also significantly different when the surgery is performed for recurrent benign or recurrent malignant disease: the injury to the nerve in such cases can be due to the difficulty in identifying and preserving the nerve encased in the scar tissue of the previous surgery (27, 29, 31, 38). These results are summarized and shortly commented in Table 4 (42, 44, 47–49).

**Table 3:** **The incidence of morbidity on the inferior laryngeal nerve according to different experiences reported in literature: the sequence is, again, progressive, starting from the series reporting the lowest incidence**.

Reference	Patients/nerves at risk	Nerve injuries (%) (transient/permanent)
Efremidou et al. (39)	932/1864	1.3/0.2°
Bergamaschi et al. (40)	1163/2010	2.9/0.3
Chiang et al. (41)	521/704	5.1/0.9
Lo et al. (42)	500/787	5.2/0.9
Thomusch et al. (43)	7266/13436	2.1/1.1°
Rosato et al. (38)	14934/n.a.	3.4/1.4
Lefevre et al. (44)	685/- (reoperations)	?/1.5
Toniato et al. (45)	504/1008	2.2*^§^
Echternach et al. (46)	1001/1365	6.6

°Only thyroidectomies for benign disease were considered; *only thyroidectomies for malignant disease were considered. ^§^Post-operative laryngoscopy not routinely performed.

**Table 4:** **Risk factors affecting the final rate of inferior laryngeal nerve injuries according to different authors**.

Reference	Patients	Study	RNL (%)	Comment
Dralle et al. (47)	16448	Retrospective, multicentric	–	Risk factors for permanent RNI: recurrent benign (4.7×) and malignant (6.7×) disease; thyroid malignancy (2×); lobectomy (1.8×)
Erbil et al. (48)	3250	Retrospective	1.8	Extended surgery (12×) and reoperations (3×) had a significant effect on the incidence of complications
Lefevre et al. (44)	685 (reoperations)	Retrospective	1.5	“Permanent complication rates were higher than those for primary thyroid resection”
Lo et al. (42)	500	Prospective	1.4	“Thyroid surgery for malignant neoplasm and recurrent substernal goiter was associated with an increased risk of permanent nerve palsy”
Shindo and Stern (49)	122 (TT + CC) Vs. 134 (TT)	Retrospective	5 vs. 10 (temp.); 0 vs. 1 (perm.)	More extended surgery on the lymph nodes is burdened by a higher rate of temporary injuries

*The cited papers are those with the highest statistical power (a high statistical power is necessary to evaluate a rare event), and this is reflected by the sequence of the citations, from the most to the less powerful ones*.

*RNL, inferior laryngeal nerve lesions; TT + CC, total thyroidectomy and central neck dissection; TT, total thyroidectomy*.

**Figure 4:**
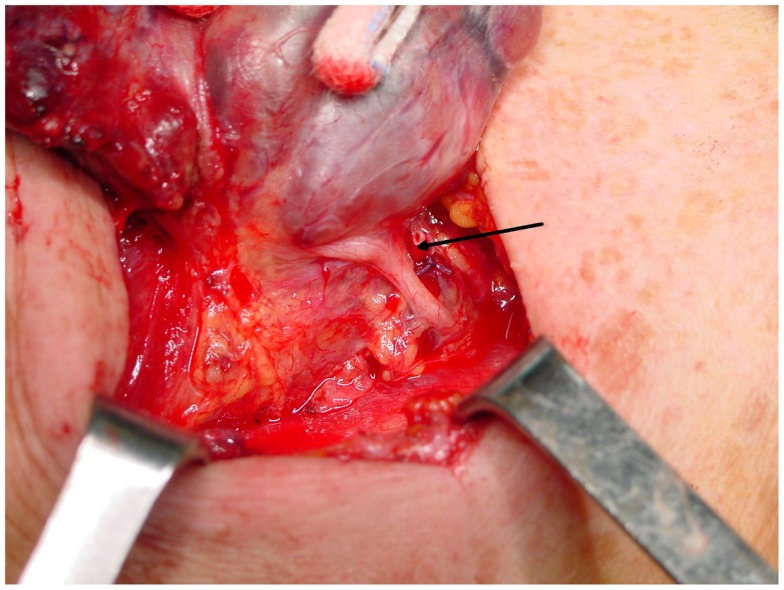
**The strict anatomical relationship between the inferior laryngeal nerve and the tubercle of Zuckerkandl**. The nerve (arrow) almost adheres to the inferior side of the tubercle, immediately before entering the laryngeal muscles.

A cornerstone of the recommended surgical technique of thyroidectomy is the active search for and identification of the RLN. It is classically identified in the Simon triangle (4), formed by the esophagus medially, carotid artery laterally and inferior thyroid artery superiorly. The tubercle of Zuckerkandl is another landmark that can be useful in identifying the RLN, since it generally lies anterior to the nerve (Figure 4) (50). Obviously, the thyroid surgeon must be aware of the possible anatomical variations of the nerve, and always search for its visual control and gentle dissection of RLN, which are imperative since possible mechanisms of injury include transection, clamping, ligation, traction, thermal injury, and ischemia. A vast majority of injuries occur because of an excessive traction on this extremely delicate structure (these lesions are those more prone to be temporary ones), and in the setting of anatomical variants, especially when an extralaryngeal bifurcation of RLN is present (4, 27, 31).

The use of intraoperative nerve monitoring (IONM) in association to visual identification of the nerve has been safely applied, even if there is lack of high-level evidence that might elucidate the true RLN sparing effect of IONM: this tool should therefore not be used as the sole mechanism for identifying and preserving the nerve, although it can be used to aid in the identification and dissection of the nerve.

In conclusion, a “safe” technique for thyroidectomy cannot be described, since every expert thyroid surgeon is burdened by a morbidity rate that cannot ever reach the 0%. Nevertheless, this rate can be “kept to a minimum” when a thorough surgical technique is followed, and the surgeon should always keep in mind that different preoperative indications correspond to a different incidence of morbidity, accordingly informing the patients about it.

## Author Contributions

All Authors (Emanuela Varaldo, Gian Luca Ansaldo, Matteo Mascherini, Ferdinando Cafiero, and Michele N. Minuto) made substantial contributions to conception and design, acquisition of data, and analysis and interpretation of data, and all participated in drafting the article and gave final approval of the version to be submitted and published.

## Conflict of Interest Statement

The authors declare that the research was conducted in the absence of any commercial or financial relationships that could be construed as a potential conflict of interest.
